# Induction of HLA-B27 heavy chain homodimer formation after activation in dendritic cells

**DOI:** 10.1186/ar2492

**Published:** 2008-08-29

**Authors:** Susana G Santos, Sarah Lynch, Elaine C Campbell, Antony N Antoniou, Simon J Powis

**Affiliations:** 1 Bute Medical School, Westburn Lane, University of St Andrews, Fife, KY16 9TS, UK; 2 Department of Immunology and Molecular Pathology, Windeyer Institute of Medical Science, 46 Cleveland St, University College London, London, W1T 4JF, UK

## Abstract

**Introduction:**

Ankylosing spondylitis (AS) is a severe, chronic inflammatory arthritis, with a strong association to the human major histocompatibilty complex (MHC) class I allele human leucocyte antigen (HLA) B27. Disulfide-linked HLA-B27 heavy-chain homodimers have been implicated as novel structures involved in the aetiology of AS. We have studied the formation of HLA-B27 heavy-chain homodimers in human dendritic cells, which are key antigen-presenting cells and regulators of mammalian immune responses.

**Method:**

Both an *in vitro *dendritic-like cell line and monocyte-derived dendritic cells from peripheral blood were studied. The KG-1 dendritic-like cell line was transfected with HLA-B27 cDNA constructs, and the cellular distribution, intracellular assembly and ability of HLA-B27 to form heavy-chain homodimers was compared with human monocyte-derived dendritic cells after stimulation with bacterial lipopolysaccharide (LPS).

**Results:**

Immature KG-1 cells expressing HLA-B27 display an intracellular source of MHC class I heavy-chain homodimers partially overlapping with the Golgi bodies, but not the endoplasmic reticulum, which is lost at cell maturation with phorbyl-12-myristate-13-acetate (PMA) and ionomycin. Significantly, the formation of HLA-B27 homodimers in transfected KG-1 cells is induced by maturation, with a transient induction also seen in LPS-stimulated human monocyte-derived dendritic cells expressing HLA-B27. The weak association of wildtype HLA-B*2705 with the transporter associated with antigen processing could also be enhanced by mutation of residues at position 114 and 116 in the peptide-binding groove to those present in the HLA-B*2706 allele.

**Conclusion:**

We have demonstrated that HLA-B27 heavy-chain homodimer formation can be induced by dendritic cell activation, implying that these novel structures may not be displayed to the immune system at all times. Our data suggests that the behaviour of HLA-B27 on dendritic cells may be important in the study of inflammatory arthritis.

## Introduction

Ankylosing spondylitis (AS) and related spondyloarthropathies (SpA) are strongly associated with the major histocompatibilty complex (MHC) class I allele human leucocyte antigen (HLA) B27. Several theories have developed to explain the link between HLA-B27 and SpA, the classical example being based on its antigen presentation function and the possibility of molecular mimicry [1]. However, the absence of a bona fide arthritogenic peptide and transgenic rat studies demonstrating a significant role in disease onset for CD4^+^, rather than CD8^+^, T cells while not ruling out a role for peptide presentation, suggests that other mechanisms may also be involved [2,3].

More recently, theories have emerged based on several non-antigen presentation properties of HLA-B27 [4]. One area of particular focus has been the demonstration of misfolding of HLA-B27 in the endoplasmic reticulum (ER), which leads to induction of the unfolded protein stress response [5]. Also, natural killer (NK) receptor recognition of non-canonical conformations of HLA-B27, in the form of heavy-chain homodimers has been reported as a potential contributor to AS development [6]. B27 homodimers were first discovered during *in vitro *MHC class I folding studies [7], and subsequently reported in cell lines, transgenic animals and patient samples [8-10]. These cell surface HLA-B27 homodimers can be recognised by NK receptors such as KIR3DL2 that do not recognise the monomeric form [11]. Enhanced numbers of NK cells and CD4+ T cells expressing these receptors have been reported in AS patients [12]. However, factors influencing the formation of HLA-B27 heavy-chain dimers remain poorly characterised.

Dendritic cells are essential to the initiation of most antigen-specific immune responses, as well as being involved in innate immune responses [13]. As such they are also pivotal to the understanding of disease and autoimmune phenotypes [14]. Recent observations into potentially abnormal interactions of HLA-B27 expressing dendritic cells with non-antigen specific T cells have brought dendritic cells into the forefront of AS research [15].

Here we show, in a human dendritic cell-like cell line and in human monocyte-derived dendritic cells, that the formation of HLA-B27 homodimers follows maturation and activatory stimuli. Our data indicates that heavy-chain dimer formation can be a relatively transitory feature induced by activation, which may impact on dendritic cell behaviour during a critical period of a developing immune response.

## Materials and methods

### Cells

The human KG-1 cell line (expressing HLA-A30, -A31, -B35 and -Cw4; ECACC, HPA Cultures, Wiltshire, UK) was maintained in Iscove's Modified Dulbecco's Medium (IMDM) (Gibco, Paisley, UK), plus 20% fetal bovine serum ([FBS] Gibco, Paisley, UK) and kanamycin (Gibco, Paisley, UK). Stable transfectants of KG-1 made with cDNA for HLA-B*2705 with and without the C-terminal sv5 epitope tag [16] were generated using the Amaxa Nucleofector (Amaxa AG., Cologne, Germany). Site-directed mutagenesis to generate mutant B27.H114D.D116Ysv5 (histidine to aspartic acid at position 114, and aspartic acid to tyrosine at position 116) was performed using Stratagene Quickchange (Stratagene, La Jolla, USA) methodology. Transfectants were selected and maintained in 1 mg/ml G418 (Geneticin, Invitrogen, Paisley, UK). KG-1 cells were differentiated/matured with 10 ng/ml phorbyl-12-myristate-13-acetate (PMA) (Sigma, Poole, UK) and 100 ng/ml ionomycin (Sigma, Poole, UK).

In agreement with the local medical school ethics committee, informed written consent was obtained from donors before blood collection. Samples were obtained from two HLA-B27-expressing individuals and two non-HLA-B27-expressing individuals, as determined by flow cytometry with fluorescein isothiocyanate (FITC) labelled-anti-HLA-B27 (VH Bio, Gateshead, UK). For primary monocyte-derived dendritic cells, peripheral blood mononuclear cells were obtained after centrifugation over Histopaque (Sigma, Poole, UK). Monocytes were allowed to adhere for two hours in RPMI-1640 medium supplemented with 10% heat inactivated FBS and kanamycin (Gibco, Paisley, UK). Non-adherent cells were then removed and fresh medium supplemented with 50 ng/ml granulocyte macrophage colony-stimulating factor (GM-CSF) and 50 ng/ml interleukin (IL)-4 was added to the culture. Dendritic cells were allowed to differentiate for four days, before treatment with 50 ng/ml lipopolysaccharide (LPS, Sigma, Poole, UK) for the indicated time periods.

### Reagents and antibodies

The following antibodies were used in this study: monoclonal anti-v5 tag (pK); W6/32 recognises folded HLA-A, B and C molecules; ME1 recognises folded HLA-B molecules; HC-10 recognises partially folded HLA-B and -C molecules; 148.3 recognises human transporter associated with antigen processing (TAP)1 [17]; anti-CD11c (Serotec, Kidlington, UK). Bodipy-ceramide (Molecular Probes, The Netherlands), goat anti-mouse HRP-conjugated secondary antibody (Perbio, Cramlington, UK) and LPS from *Salmonella enteritidis *(Sigma, Poole, UK) were also used.

### Flow cytometry and immunofluorescence

Cells were resuspended in PFN (phosphate buffered saline [PBS], 2% FBS, 0.1% sodium azide), stained with the indicated antibodies and FITC-labelled second stage. Mouse immunoglobulins were used as negative controls to define background staining. Samples were analysed on a FACScan (BD Biosciences, Oxford, UK) using BD Biosciences CellQuest software. For immunofluorescence microscopy cells were fixed in 2% formaldehyde in PBS, blocked with 1% BSA in PFN containing 0.2% saponin, stained with antibodies in PFN with 0.2% saponin, and stained with FITC-labelled second stage. The Golgi marker Bodipy TR C5-ceramide (Molecular Probes, Leiden, The Netherlands) was used according to the manufacturer's instructions. Stained cells were mounted with Dapi (4',6-diamidino-2-phenylindole) containing Vectashield (Vector Laboratories, Peterborough, UK). Deconvolution microscopy was performed with a DeltaVision Restoration Imaging System (Applied Precision, Issaquah, USA). A z-series of 15 to 45 images at 0.35 μm intervals was captured and processed using constrained interactive deconvolution via SoftWoRx 3.0 software (Applied Precision, Washington, USA). Further image analysis including volume sections were generated using Image J open source software.

### Western blotting, biotinylation and immunoprecipitations

Cell lysates were prepared by pre-treating cells with 10 mM N-ethyl maleimide (NEM) in PBS on ice for 10 minutess, and then lysed in 1% NP40 lysis buffer with 10 mM NEM and 1 mM phenylmethanesulphonylfluoride (PMSF). Lysates were centrifuged at 14,000 rpm for 10 minutes. Protein was quantified using Bradford reagents (Sigma, Poole, UK).

For biotinylation, cells were pre-treated with 10 mM NEM on ice for 10 minutes before being labelled with 0.1 mg/ml Sulfo-NHS-biotin (Sigma Poole, UK) for 10 minutes on ice. Free biotin was quenched using Tris buffer (TBS) with 5% FBS, samples were washed twice in TBS, followed by lysis as above. Pre-cleared lysates were incubated with streptavidin-agarose (Sigma, Poole, UK) beads for 25 minutes. Beads were washed three times with lysis buffer, and resuspended in 20 μl non-reducing sample buffer.

Samples were run on 8% sodium dodecyl sulphate polyacrylamide gel electrophoresis (SDS-PAGE), transferred to nitrocellulose membrane and probed with the indicated antibodies or streptavidin-HRP (Sigma, Poole, UK) and signals monitored by chemiluminescence (Perbio, Cramlington, UK). Two-dimensional gel analysis was performed as previously described [18], followed by immunoblotting as above. Pulse-chase analysis was performed by labelling cells with 3.7 MBq Trans-label (MP Biomedicals, Solon, USA), followed by lysis and immunoprecipitation with relevant antibodies and Protein G beads (Sigma, Poole, UK). Immune complexes were digested with 2.5 mU endoglycosidase H (Roche, West Sussex, UK) for one hour at 37°C prior to SDS-PAGE.

## Results

### Distribution of HLA-B27 transfected KG-1 cells

**Figure 1:**
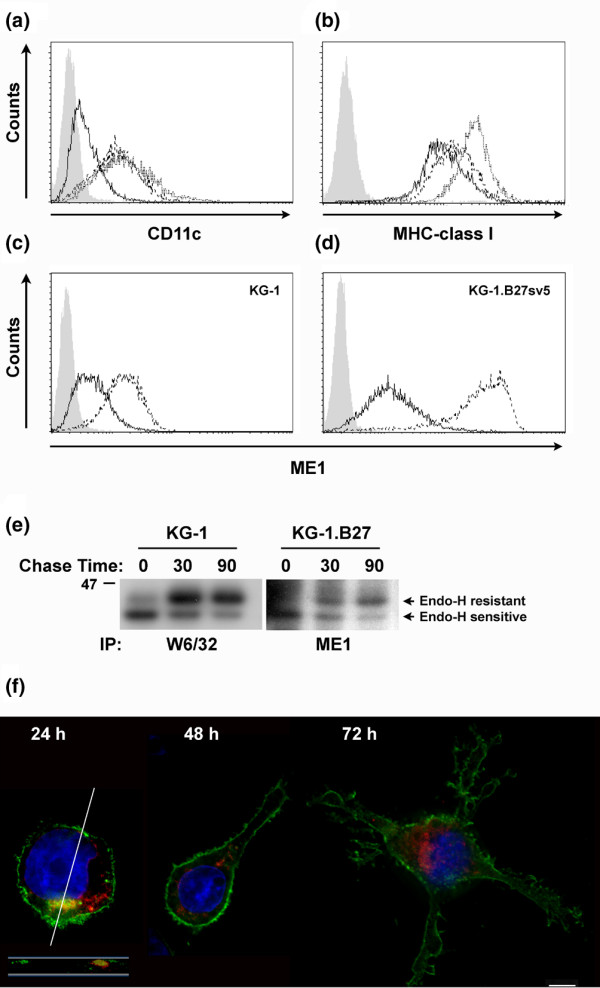
Expression of HLA-B27 in KG-1 cells. Unstimulated KG-1 cells (solid line) and KG-1 cells stimulated with PMA and ionomycin for 24 hours (dashed line) or 72 hours (dotted line), were surface stained for **(a) **DC markers CD11c and **(b) **W6/32 for MHC class I. **(c) **KG-1 and **(d) **KG-1.B27sv5 cells, unstimulated (solid line) or stimulated with PMA and ionomycin for 24 hours (dashed line), were surface stained with ME1, recognising HLA-B alleles. The grey curves indicate second stage anti-mouse FITC alone. **(e) **KG-1 and KG-1.B27 cells were metabolically labelled with ^35^S-Trans label, and cell lysates immunoprecipitated at the indicated time points with W6/32 or ME1 antibodies, and digested with endoH. **(f) **KG-1.B27 cells were differentiated with PMA and ionomycin for 24, 48 or 72 hours, and stained with ME1 (green) and the Golgi dye Bodipy Ceramide (red). The white bar represents 5 μm. A panel below the 24 hour image displays a red and green merged volume section, from the region indicated by the white line.

In agreement with a previous report [19], with stimulation with PMA and ionomycin for 24 hours and 72 hours, KG-1 cells up-regulated cell surface CD11c, MHC class I (Figures 1a, b), and CD83 (not shown). KG-1, which do not express endogenous HLA-B27, were transfected with non-tagged and epitope-tagged versions of HLA-B*2705 cDNA. Cells expressing epitope-tagged HLA-B27 (KG-1.B27sv5) up-regulated cell surface expression (Figures 1c, d), as did transfectants expressing non-epitope tagged HLA-B27 (not shown).

Note that in untransfected KG-1 cells the anti-HLA-B mAb ME1 weakly recognises the endogenous HLA-B35 allele (Figure 1c). The increase detected in HLA-B27 surface expression (Figure 1d) therefore occurred despite the cDNA being under a non-endogenous (CMV) promoter. This may reflect the increase in expression of components of the MHC class I assembly pathway, such as ERp57 and tapasin, which we have previously shown to increase with KG-1 maturation [20].

To determine the assembly kinetics of endogenous HLA class I alleles in KG-1 cells in comparison to the expressed HLA-B27 cDNA, we performed pulse-chase analysis. In KG-1.B27 cells, HLA-B27 molecules acquire endoglycosidase H resistance (generally indicating exit of folded molecules from the ER) with similar kinetics to the general MHC class I pool recognised by W6/32 in KG-1 cells (Figure 1e). ME1 immunoprecipitation of untransfected KG-1 cells did not reveal detectable signal (not shown), in accordance with the relatively weak flow cytometric staining in Figure 1c. Taken together, our data suggests that the assembly of HLA-B*2705 in KG-1 cells appears similar to recent data studying the assembly of multiple HLA-B27 alleles, wherein various subtypes of HLA-B27 acquired 50% endoglycosidase H resistance within a range of 14 minutes (HLA-B*2706) to 52 minutes (HLA-B*2705) [21].

Some subpopulations of dendritic cells have the capacity to store MHC class I molecules in an intracellular compartment [22]. Similarly, immature KG-1 cells have previously been shown to retain some MHC class I molecules in the Golgi [19], a result we confirm also occurs with HLA-B27 (Figure 1f), where cells stained with ME1 (green) and the Golgi specific stain Bodipy-ceramide (red) show significant co-localisation. The same results were observed with specific B27-FITC antibody reagents on KG-1.B27sv5 cells (not shown). No significant overlap of HLA-B27 was observed with the ER-resident chaperone calreticulin (not shown). Redistribution of the HLA-B27 signal to the cell surface occurs, as expected, on maturation (Figure 1f), with strong staining of dendritic cell-like structures. Thus, HLA-B27 molecules expressed in KG-1 dendritic cell-like cells overall display behaviour similar to other MHC class I molecules previously reported in this cell line [19], confirming it as a suitable model for the study of HLA-B27 in dendritic cell-like cells.

### Association of HLA-B27 molecules with TAP in KG-1 cells

**Figure 2:**
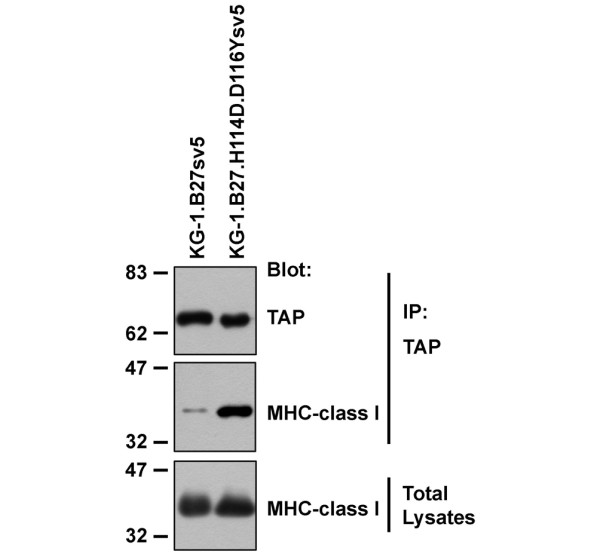
Association of HLA-B27 with TAP in KG-1 cells. KG-1.B27sv5 and KG-1.B27.H114D.D116Ysv5 cells were stimulated for 24 hours, lysed in digitonin-containing buffer and immunoprecipitated with anti-TAP1 antibodies conjugated to Protein G beads. Samples and cell lysate controls were resolved on sodium dodecyl sulphate polyacrylamide gel electrophoresis (SDS-PAGE) and immunoblotted with the indicated antibodies. Molecular mass markers are shown in kDa.

The MHC class I specific accessory molecule tapasin forms part of the MHC class I peptide loading complex and assists in optimising the pool of peptides bound by MHC class I molecules [23]. However, HLA-B27 can be expressed efficiently in the absence of tapasin [24], even though it is loaded with a suboptimal peptide cargo [23], suggesting a partial non-reliance on TAP-association for peptide-loading. HLA-B27, when expressed in tapasin-deficient cells, forms increased amounts of HLA-B27 homodimers [9]. To assess the ability of HLA-B27 to interact with tapasin in our KG-1 dendritic cell model system, we determined the ability of HLA-B27 to co-immunoprecipitate with TAP in digitonin lysates of our transfected KG-1.B27 cells. In addition we compared it with a mutant of HLA-B27 in which residues at positions 114 and 116 were mutated to mimic the less disease-associated allele HLA-B*2706 (mutant termed H114D.D116Y). Residue 114 has been implicated in determining tapasin, and therefore TAP association [24]. As shown in Figure 2, HLA-B27 was detected in association with TAP, but in significantly reduced amounts compared with mutant KG-1.B27.H114D.D116Y. Thus in KG-1 dendritic cell-like cells HLA-B*2705 interacts relatively weakly with TAP.

### Induction of HLA-B27 heavy chain-homodimers after dendritic cell stimulation

We then determined the ability of the HLA-B27-expressing KG-1 cells to form the heavy-chain homodimer structures implicated in NK-receptor recognition events [6]. Cells were treated with PMA/ionomycin and whole-cell lysates tested at 0, 24 and 72 hours for dimer formation by immunoblotting with the HLA-B reactive monoclonal antibody (mAb) HC10 and epitope tag-specific mAb Pk. MHC class I signal increased at 24 and 48 hours after stimulation in both cell lines, especially in the transfected cells, mirroring our flow cytometry data shown in Figure 1d.

**Figure 3:**
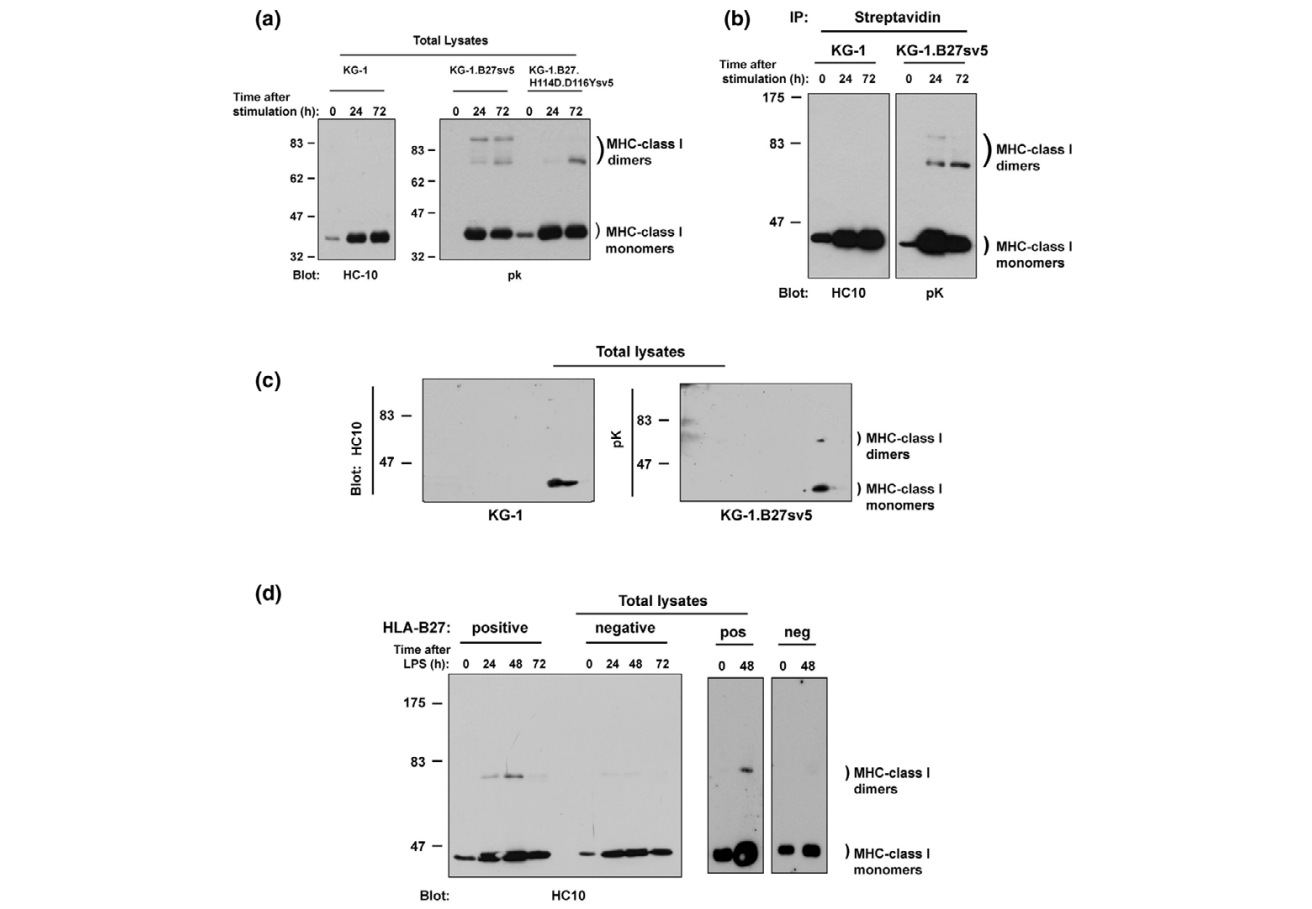
Induction of HLA-B27 heavy-chain dimers in KG-1 cells and human dendritic cells. KG-1, KG-1.B27sv5 and KG-1.B27.H114D.D116Ysv5 cells were stimulated with PMA and ionomycin for the indicated times **(a) **before preparation of cell lysates or surface biotinylation and **(b) **pulldown with streptavidin-agarose beads. Immunoblots were probed with HC10 or anti-tag pK. **(c) **In 24-hour stimulated KG-1 and KG-1.B27sv5, cell lysates were resolved under non-reducing conditions by two-dimensional gel electrophoresis, followed by immunoblotting with HC10 or pK. MHC class I dimers and monomers are indicated. **(d) **In human monocyte-derived dendritic cells were generated from peripheral blood monocytes of two HLA-B27-positive individuals and two negative controls, before being stimulated for 0, 24, 48 or 72 hours with lipopolysaccharides. Cells lysates were resolved under non-reducing conditions and immunoblotted for MHC-class I heavy chain (HC-10). Class I dimers and monomers are indicated. Molecular weight markers are shown in kDa.

Heavy-chain dimer structures were detected in KG-1.B27 cells after 24 hours of stimulation, at which point multiple bands in the 80 kDa region were visible (Figure 3a), and at 72 hours in KG-1.B27.H114D.D116Y cells, in which the higher of the bands detected in KG-1.B27 cells was only weakly visible. The appearance of the HLA-B27 dimer structures later in KG-1.B27.H114D.D116Y cells may reflect its stronger association with tapasin/TAP (Figure 2).

To determine whether any of these structures were also expressed at the cell surface, we performed cell surface-specific biotinylation, followed by streptavidin pulldown and immunoblotting for MHC class I molecules [11]. Figure 3b shows that the lower of the 80 kDa-region bands was most highly represented at the cell surface in KG1-B27 cells. To confirm this 80 kDa species as a B27 heavy-chain homodimer, we performed immunoblotting of cell lysates separated by two-dimensional electrophoresis. A spot resolved at 80 kDa directly above the HLA-B27 monomer in transfected KG-1 cells (Figure 3c), which was the result expected for two associated heavy chains possessing an identical isoelectric point of the HLA-B27 monomer. Overlaying gels confirmed this spot as having similar migration characteristics as the lower of the dimer-region bands seen in Figures 3a and 3b. We were unable to detect any other significant spots that would allow us to determine the nature of the other bands present in HLA.B27 transfected KG-1 lysates.

The above observations suggested that HLA-B27 heavy-chain homodimers were a transient structure, reliant on the activation status and/or maturation of KG-1 cells for their formation. Since most studies of HLA-B27 have focused on activated cells or cells lines, we wished to determine if our observations might also extend to HLA-B27 expressed endogenously in primary human dendritic cells. We therefore differentiated peripheral blood monocyte-derived dendritic cells from one individual expressing HLA-B27, and one control non-B27-expressing individual. The dendritic cells were stimulated via TLR4, using LPS and whole cell lysates prepared at times up to 72 hours after stimulation. Similar to the results for KG-1 cells, the primary HLA-B27 expressing dendritic cells displayed the induction of dimer formation on stimulation. We repeated these observations in two further dendritic cell samples stimulated with LPS for 48 hours. Of significant interest, in the extended maturation cultures, heavy-chain dimer levels peaked at 48 hours, declining thereafter (Figure 3d). Our results show, for the first time, that B27 dimers can be detected as a transient population in dendritic cells and are dependent on their activation status.

## Discussion

Most immune responses, both innate and adaptive, involve the activation of multiple immune cell types, as a result of which many up-regulate the expression of components of the antigen presentation pathway, including MHC class I molecules. We therefore set out to determine whether dendritic cells expressed HLA-B27 dimers, and to what extent cell activation could induce HLA-B27 dimers in dendritic cells, which are crucial cells for most immune responses, and have recently been implicated in AS [15]. In this study we have demonstrated the formation of HLA-B27 heavy-chain dimers in a transfected dendritic cell-like cell line, and in HLA-B27-positive monocyte-derived human dendritic cells.

Significantly, dimers were essentially undetectable in unstimulated cells, but usually appeared within 24 hours of activation. Similarly, in macrophages from HLA-B27-expressing disease-prone transgenic rats, HLA-B27 dimer-like structures are readily detected after stimulation with interferon (IFN)-γ [5]. Thus, it is possible that these structures may only appear during an active immune response. However, since we detected dimers concomitantly with an increase in overall levels of HLA-B27 heavy-chain dimers, it may be that dimers are normally present at levels below our current detection level, a limitation that we cannot at this stage formally exclude. Nonetheless, it is possible that the immune system, since it relies on many receptor-based interactions which involve the clustering of ligands including MHC class I molecules [25], may likewise not be able to see very low levels of dimer structures, and may therefore itself rely on cell activation to detect them. This observation could have an impact on the study of HLA-B27-associated arthritis. For example, HLA-B27-associated reactive arthritis usually develops after the significant immunological challenge of a bacterial gut infection. Similarly, the disease-prone HLA-B27 transgenic rat model is essentially disease free when kept in specific pathogen-free conditions, and only succumbs to disease when removed from these conditions [26]. Both of these could be interpreted as sequelae to the induction of large numbers of HLA-B27 heavy-chain dimers on antigen-presenting cells, although it does not explain why other significant immune challenges, such as viral infections, are not seen to similarly trigger inflammatory arthritis.

In the case of the human monocyte-derived dendritic cells, we also observed a decrease in dimer formation between 48 and 72 hours after activation. Recent *in situ *studies in the rat show that dendritic cells from the intestine migrate to mesenteric lymph nodes within 24 to 48 hours [27]. This could result in the temporal exposure of HLA-B27 dimer structures within the lymph node, where many of the crucial interactions that determine immune responses occur.

The fact that increased expression of MHC class I molecules may predispose to heavy-chain dimer formation may be relevant in other alleles as well as HLA-B27. We detected very low quantities of dimer-sized bands in the HLA-B27-negative dendritic cell cultures (Figure 3d), and are currently investigating the nature of these bands, which may represent a novel MHC class I structure formed in non-HLA-B27-expressing cells (SL and SJP, unpublished observations). We have previously observed transient heavy-chain dimer-like structures in activated peripheral blood lymphocytes [28]. Furthermore, in studies of the tapasin-deficient .220 cell line, when restored with tapasin and high levels of HLA-B8 we also detect the presence of dimer-like structures (SL and SJP, unpublished observations). How such dimers form in the absence of the unpaired cysteine at position 67 in the peptide-binding groove, which has been shown to be involved in HLA-B27 dimer formation [7], remains to be determined, although contributions from non-covalent interactions in high molecular weight HLA-B27 structures have been reported [29].

Although we have not included a significant number of human samples in this present study, nor samples from patients with actively defined AS, our data does suggest it would be of interest to determine the burden of dimer structures that may exist in non-activated and activated cells of different lineages, in both the rat transgenic model and in human cells from HLA-B27 healthy controls and patients with AS.

## Conclusion

In summary, our data indicate that detectable HLA-B27 heavy-chain dimer formation may be induced in important cell types such as dendritic cells only on receiving an activatory stimulus. A comprehensive study of heavy-chain dimer formation in HLA-B27-positive individuals, both with and without AS, and its correlation with dendritic cells and other immune cell activation, is currently lacking, but would probably provide further insights into the potential availability of heavy-chain dimers as targets for interaction with T and NK cell receptors.

## Abbreviations

AS = ankylosing spondylitis; ER = endoplasmic reticulum; FBS = fetal bovine serum; HLA = human leucocyte antigen; IFN = interferon; IL = interleukin; LPS = lipopolysaccharide; MHC = major histocompatibility complex; NEM = n-ethyl maleimide; NK = natural killer cell; PBS = phosphate buffered saline; SDS-PAGE = sodium dodecyl sulphate polyacrylamide gel electrophoresis; SpA = spondyloarthropathies; TAP = transporter associated with antigen processing; TBS = Tris buffer.

## Competing interests

The authors declare that they have no competing interests.

## Authors' contributions

SS generated the transfectants and performed experimental work in the study, designed experiments and drafted the manuscript. SL and EC carried out immunoblotting work in the study and drafted the manuscript. AN performed site-directed mutagenesis and was involved in the drafting of the manuscript. SP designed experiments and wrote the manuscript.
